# Evaluation of cortisol and telomere length measurements in ethnically diverse women with breast cancer using culturally sensitive methods

**DOI:** 10.1007/s12687-016-0288-y

**Published:** 2017-01-03

**Authors:** Julio Ramirez, May Elmofty, Esperanza Castillo, Mindy DeRouen, Salma Shariff-Marco, Laura Allen, Scarlett Lin Gomez, Anna María Nápoles, Leticia Márquez-Magaña

**Affiliations:** 1 Health Equity Research Laboratory, San Francisco State University, 1600 Holloway Avenue, San Francisco, CA 94132 USA; 2 Cancer Prevention Institute of California, 2201 Walnut Avenue, Suite #300, Fremont, CA 94538 USA; 3 Stanford Cancer Institute, Lorry Lokey Building/SIM 1, 265 Campus Drive, Ste G2103, Stanford, CA 94305 USA; 4 Center for Aging in Diverse Communities, Division of General Internal Medicine, Department of Medicine and the Helen Diller Family Comprehensive Cancer Center, University of California San Francisco, San Francisco, USA

**Keywords:** Biospecimens, Ethnic minority populations, Cortisol levels, Telomere length

## Abstract

The under-representation of ethnic minority participants, who are more likely to be socially disadvantaged in biomedical research, limits generalizability of results and reductions in health disparities. To facilitate investigations of how social disadvantage “gets under the skin,” this pilot study evaluated low-intensity methods for collecting hair and saliva samples from multiethnic breast cancer survivors (*N* = 70) and analysis of biomarkers of chronic stress (cortisol levels) and biological age (telomere length). Methods allowed for easy self-collection of hair (for cortisol) and saliva (for telomere lengths) samples that were highly stable for shipment and long-term storage. Measuring cortisol in hair as a biomarker of chronic stress was found to overcome many of the limitations of salivary cortisol measurements, and the coefficient of variation obtained using an ELISA-based approach to measure cortisol was within acceptable standards (16%). Telomere length measurements obtained using a qPCR approach had a coefficient of variation of <10% when the DNA extracted from the saliva biospecimens was of sufficient quantity and quality (84%). The overall response rate was 47%; rates were 32% for African-Americans, 39% for Latinas, 40% for Asians, and 82% for non-Latina Whites. Self-collection of hair and saliva overcame cultural and logistical barriers associated with collection of blood. Results support the use of these biospecimen collection and analysis methods among ethnically diverse and disadvantaged populations to identify biopsychosocial pathways of health disparities. Our tools should stimulate research to better understand how social disadvantage “gets under the skin” and increase participation of ethnic minorities in biomedical research.

## Introduction

Despite recent progress, racial/ethnic disparities in health remain substantial in the USA (National Healthcare Quality and Disparities Report 2014). Progress toward elimination of these disparities is hindered in part by limited inclusion of ethnic minorities in biomedical research studies (Oh et al. 2015), thereby limiting the generalizability of the results. Among women diagnosed with breast cancer, racial/ethnic disparities in outcomes persist and in some cases continue to grow (Curtis et al. 2008; Hunt et al. 2014). For instance, African-American (AA) women die of breast cancer at a much higher rate than White women (Ries et al. 2003), a disparity that remains when disease stage, tumor type, and access to care are taken into account. These observations suggest that breast cancer disparities may be a function of social/environmental factors (Wu et al. 2013; Shariff-Marco et al. 2015; Tao et al. 2015; Keegan et al. 2015). Furthermore, socially disadvantaged neighborhoods contribute to elevated stress levels of residents and may play a role also (Bradley et al. 2002; Baquet and Commiskey 2000; Whitman et al. 2011; Ansell et al. 2009; Hunt et al. 2014).

The biological manifestation of chronic stress depends on interactions among the experience of stressors, available support or stress buffers, and individuals’ personality and coping mechanisms (Chen and Miller 2013; Hertzman and Boyce 2010; Braveman et al. 2011). Studies suggest that chronic stress contributes to racial/ethnic disparities in a multitude of health outcomes, including breast cancer survival (De Brabander and Gerits, 1999; Chida et al. 2008; Andersen et al. 2008). It is apparent that chronic stress can lead to negative health outcomes through a number of different mechanisms including altered neuroendocrine signaling, immune suppression, increased oxidative stress, and unhealthy behaviors that are associated with comorbid conditions (i.e., hypercholesterolemia, hyperglycemia, and hypertension) (Reiche et al. 2004; Lin et al. 2012). These mechanisms may exert independent effects on mortality, as well as cause telomere erosion (Shalev et al. 2013; Wolkowitz et al. 2010; Lin et al. 2012). Telomeres are DNA-protein complexes located at the end of chromosomes that protect them from engaging in genetic rearrangements and thus are responsible for maintaining integrity of the genome. Telomeric DNA is gradually lost each time a cell divides, and when telomeres reach a critically short length, aging cells proceed into apoptosis and death (Blackburn et al. 2006). Thus, telomere length is considered a robust indicator of biological age and overall health (Wolkowitz et al. 2010). Furthermore, shortening of telomeres may hasten cancer progression, as well as increase risk of recurrence (Artandi and DePinho 2010; Avigad et al. 2007; Willeit et al. 2010; Gramatages et al. 2014).

Measures of chronic stress have consisted usually of salivary cortisol or immune components in blood plasma. Telomeres have been most often assessed with white blood cells. However, these measures have some limitations when applied in studies of cancer health disparities. A limitation of traditional measures of cortisol is that diurnal rhythms and acute responses of cortisol in saliva and urine may not be representative of long-term exposure to stress (Russell et al. 2012), which may be an especially critical risk factor for vulnerable populations. Cortisol levels in blood or saliva are inherently subject to diurnal variation necessitating that samples be collected at the same time every day for reliable comparison across individuals. Measuring cortisol in urine requires 24-h collection and immediate refrigeration, as is the case for blood and saliva. Telomere length (TL) is most commonly determined using DNA that is extracted from blood cells. However, the collection of blood requires immediate refrigeration of this biospecimen, creating additional obstacles, including increased costs of biospecimen collection. The collection of blood is invasive requiring a needle and syringe, and participants must travel to a research/medical facility to donate or allow a certified phlebotomist into their home.

These biospecimen collection approaches may place a disproportionately high burden on already taxed vulnerable ethnic minority groups, thus may lack acceptance among these groups (Dang et al. 2014). For example, compared to Whites, African-Americans have lower response rates for blood collection for research studies (Bussey-Jones et al. 2010; McQuillan et al. 2003; Boulware et al. 2002). Alternative measures of chronic stress and biological age that are sensitive to longer-term exposures and may be more acceptable to ethnic minorities are needed, to better understand the role of social disadvantage and increased risk of cancer and associated outcomes.

Herein, we report the results of a pilot study that examined alternative approaches to the collection of hair and saliva biospecimens and measurement of biomarkers of chronic stress and biological aging that may be easier to collect, transport, and store. We also describe the optimization of molecular assays for analyzing these biospecimens to quantify cortisol levels in hair and telomere length in saliva. Biospecimen collection was conducted with participants in the Equality in Breast Cancer Care study (Quach et al. 2012), a multiethnic, multi-language population-based study of breast cancer survivors that examined discrimination in the health care context. Our aim was to develop tools for self-collection of hair and saliva biospecimens and methods for their analysis that would facilitate the participation of ethnic minorities in biomedical research that seeks to improve our understanding of how social disadvantage “gets under the skin.”

## Materials and methods

### Recruitment

Participants for this pilot study were drawn from participants in the Equality in Breast Cancer Care (EBCC) study who had agreed to participate in future research (more than 90% of 522 EBCC participants). EBCC participants were identified from the population-based Greater Bay Area Cancer Registry (GBACR, part of the NCI-funded Surveillance, Epidemiology, and End Results (SEER) program) using the following eligibility criteria: residence (at time of diagnosis and interview) in one of the San Francisco Bay Area counties (San Francisco, Contra Costa, Alameda, San Mateo, and Santa Clara), age 20 or older at diagnosis, diagnosed with first-primary invasive stages I–IV breast cancer between 2006 and 2009, and alive at the time of interview (2011–2012). Women who had experienced a recurrence were ineligible.

EBCC was a five-year study using mixed methods to develop, test, and implement a multi-language survey to address how discrimination in the health care context impacts disparities in breast cancer treatment and quality of life (Quach et al. 2012). New structured survey items were created to reflect many of the major themes derived from the qualitative phase and pretested in multiethnic respondents using cognitive interview methods (Quach et al. 2012). In 2011–2012, the 1-h interviews were completed with a population-based, multiethnic cohort of 522 breast cancer patients. Although response rates varied from 30 to 40% across racial/ethnic groups due in part to competing breast cancer studies that were recruiting participants from the GBACR, EBCC participants were similar to eligible cases from the GBACR. Telephone interviews were conducted in English, Spanish, Chinese, or Tagalog and asked about various forms of discrimination, coping, stress, social support and social networks, and quality of life. Clinical and follow-up data were extracted from the GBACR. Approval for human subject research was obtained from the Institutional Review Boards at the California State Committee for Protection of Human Subjects and Cancer Prevention Institute of California (CPIC).

### Collection of biospecimens

Biospecimen collection occurred in 2014. EBCC participants who had previously agreed to be contacted for follow-up studies and were not being invited to participate in other studies were sent an initial contact letter in English, Spanish, Chinese, or Tagalog (depending on their preferred language) explaining the study and indicating that they would be contacted by telephone to assess their interest in the pilot study, which involved donation of a hair and saliva sample. Follow-up telephone calls were made by language-matched recruiters to prospective participants to assess their interest in participating in the study. The recruitment team was trained on the potential need to clarify misconceptions and address knowledge gaps regarding use of biospecimens in research that have been found previously in multiethnic groups (Dang et al. 2014). For example, they were well prepared to answer basic scientific questions about the reasons for collection of the specific types of biospecimens needed, methods for collecting these, how they would be analyzed, and who would have access to them. The non-invasive nature of biospecimen collection was stressed.

Due to resource constraints, a biospecimen collection kit was mailed only to respondents who agreed by telephone to provide biospecimens. Because of cost limitations (this was a pilot study), quotas were set at 25 biospecimens each for 4 ethnic groups: African-Americans, non-Latina Whites, Latinas, and Asians, for a total of 100 samples. The kit included instructions for self-collection of biospecimens, an index card, a saliva collection tube, and a postage-paid, self-addressed envelope for return of biospecimens. Written instructions for self-collection of biospecimens were provided in low-literacy English, Chinese, Spanish, or Tagalog and included illustrations to increase understanding of these instructions. For hair collection, participants were instructed to cut a pencil-width section of hair from the base of the skull and use scotch tape to attach the end of the hair closest to the scalp to an index card provided in the self-collection kit. Each index card had a unique numerical identifier to link the hair biospecimen to the participant from which it was obtained. The literature suggests that hair maintains a good record of cortisol levels over time; 10 mg of hair sampled approximately 2–3 cm from the scalp should represent 2–3 months of growth and cortisol exposure (Koren et al. 2008).

For saliva collection, participants were asked to spit into a commercially available saliva collection tube (DNA Genotek), which was labeled with the same unique identifier. Participants were asked to provide saliva samples first thing in the morning, upon getting up, before doing anything else. Finally, participants were asked to return both the index card with the attached hair and the tube containing stabilized saliva to CPIC, along with a short survey with questions regarding the use of hair dye and corticosteroids, using the provided self-addressed envelope.

### Hair cortisol assay

#### Extraction of cortisol from hair

Hair biospecimens that were self-collected by study participants were maintained in sealed envelopes at room temperature until processed. The protocol used for processing hair for cortisol analysis was based on the work of various expert groups (Sauvé et al. 2007; Yamada et al. 2007; Kirschbaum et al. 2009; Skoluda et al. 2012; Manenschijn et al. 2011; D’Anna-Hernandez et al. 2011). For each sample, hair from the scalp end was cut into small pieces about a millimeter in length using small surgical scissors. Ten milligrams of the snipped hair was transferred into a disposable glass vial (1.0 DR, VWR) and washed in methanol three times to remove surface contaminants. After the last wash step, 1 ml of methanol was added to each vial and capped to minimize evaporation. Capped vials were then incubated overnight (~16 h) in a water bath at 52 °C with constant shaking (C76 water bath shaker, New Brunswick Scientific) to extract the cortisol contained within the medulla of the hair (Sauvé et al. 2007; Van Uum et al. 2008; Yamada et al. 2007; Kalra et al. 2007). Approximately 0.8 mL of supernatant was then transferred to a sterile 1.5 ml microcentrifuge tube for evaporation. The supernatant was evaporated at 38 °C under vacuum conditions (CentriVap concentrator, Labconco), and dried samples were then reconstituted in phosphate buffered saline (PBS) at pH 7.2.

#### Analysis of cortisol levels

We assessed cortisol levels in hair as a measure of long-term exposure (2–3 months of hair growth) using an immunoassay (ELISA) originally developed for cortisol measurements in saliva (ALPCO, Inc). Hair sampling and analysis were performed in triplicates from a single extraction. We used a four-parameter linear regression analysis (Gen 5 version 2.04) to determine the concentration of cortisol in nanogram per milliliter before being corrected for the individual hair weight of the sample. The limit detection of our ELISA assay was ~1 ng/ml, with a 95% confidence limit. The resulting hair cortisol concentration was then converted into pg/mg using the following conversion formula:

$$ \frac{\left[\left(\mathrm{ng}/\mathrm{mL}\right)\times \left(0.150\mathrm{mL}\right)\times \left(1\mathrm{mL}/0.8\mathrm{mL}\right)\right]}{\left(10\mathrm{mg}\times 1000\right)} $$

This equation includes the carefully measured weight (10 mg) and the volume of the reconstituted hair sample (0.150 mL) used to quantify cortisol exposure. The correction factor accounts for the initial methanol volume (1 mL) prior to the extraction step and the portion used (0.8 mL) during the evaporation step (Dr. Xiaoling Song, by communication).

### Telomere length assay

#### Extraction of DNA from cheek cells in saliva

Genomic DNA (gDNA) was extracted from buccal cells with the QiaAmp DNA blood mini kit (Qiagen) according to the manufacturer’s recommendations and was used to establish and validate the telomere length assay. The quantity and quality of the extracted gDNA was determined using the Nanodrop 2000 spectrophotometer (Thermo Scientific). The concentration of the extracted gDNA was based on absorbance at 260 nm, and the ratio of the absorbance at 260 and 280 nm (A260/280) was used to determine its purity. The quality of the gDNA was also determined by visualizing it on a 1% agarose gel stained with ethidium bromide to verify integrity. Genomic DNA was extracted from 70 biospecimens, but only gDNA samples having 260/280 ratios close to 1.7 were used for telomere length measurements. All gDNA samples were maintained at −20 °C for long-term storage.

#### Analysis of telomere length

The relative telomere length in gDNA isolated from saliva samples was determined by quantitative polymerase chain reaction (qPCR) using the T/S ratio method (Cawthon 2002), which provides a relative measure of average telomere length from gDNA by quantifying the amount of telomere repeats (T) across all chromosomes relative to the amount of a single-copy gene (S). The average telomere length is then determined as a function of the single-copy gene hemoglobin. Triplicate qPCR reactions were run on the Applied Biosystems Real-Time PCR System model 7300 (Applied Biosystems). Sample concentration was determined using a standard curve method prepared by threefold serial dilutions of a reference gDNA sample between 0.3 and 81 ng. The Applied Biosystems 7300 Real-Time PCR System software was used to convert cycle threshold (Ct) to nanograms of telomere (T) and reference gene (S). An average of the triplicate measures was used to calculate the T/S ratio by dividing the average amount of telomere product by the average amount of product obtained for the single-copy gene. Each qPCR experiment included negative controls and positive controls (Aldevron gDNA).

Amplification of telomere repeats required 1× Power SYBR Green Master Mix (Life Technologies), 0.1 and 1.0 μM of telomere-specific forward tel1b [5′-CGGTTT (GTTTGG)5GTT-3′] and reverse [5′-GGCTTG (CCTTAC)5CCT-3′] primers respectively, and 20 ng of gDNA in a total reaction volume of 20 μl final. The thermal cycling profile used for amplification of telomere repeats (T) consisted of: denature at 95 °C for 10 min followed by 30 cycles of 95 °C for 15 s and annealing/extension at 54 °C for 60 s, with fluorescence data collection. The amplification of a single-copy gene (hemoglobin) required 1× Power SYBR Green Master Mix (Life Technologies) forward [5′ GCTTCTGACACAACTGTGTTCACTAGC-3′] and reverse primers [5′-CACCAACTTCATCCACGTTCACC-3′] used at a 0.4 and 1 μM final concentration, respectively, and 20 ng of gDNA in a 20-μl final reaction volume. The cycling profile for the single-copy gene (S) consisted of denature at 95 °C for 10 min followed by 35 cycles of 15 s at 95 °C, annealing/extension at 58 °C for 60 s, with fluorescence data collection. The collected fluorescence data was used to calculate the relative telomere length (*T*/*S*) as previously described (Cawthon 2002; Lin et al. 2010).

## Results

### Accrual of biospecimens

First, investigators began with the list of 470 women who participated in the EBCC study who agreed to be recontacted. Of the 470 EBCC study participants who agree to be recontacted, a list of 203 women was stratified by race/ethnicity, after determining that cases were available for the study, that is, ruling out cases that were already being sampled for another study, per registry policy. The sampling frame for the study consisted of these 203 participants who were sent an initial contact letter for the biospecimen collection pilot study, followed by telephone call attempts. These included 25 African-American, 50 non-Latina White, 42 Latina, and 86 Asian women with breast cancer. The number of African-American women who could be sampled was particularly small because there were three other studies sampling African-American women at the time this study was conducted.

Of the 203 women who were mailed an initial contact letter, 70 (35%) of these agreed to provide any biospecimen (hair only, saliva only or both), 80 (39%) refused, 22 (11%) were not sent a collection kit by the end of the study because the sample quota of 20 was reached (almost all were non-Latina Whites), 19 (9%) were unable to be reached by telephone, 7 (3%) were deceased, and 5 (3%) were ineligible because they had cancer in a site other than breast cancer or were in treatment due to a recurrence (Table 1). Both hair and saliva samples were received from 62 women (37% were Asian, 36% were White, 21% were Latina, and 6% were African-American); an additional 7 women donated only saliva (5 were Asian and 2 were Africa- American) and one White woman donated hair only (total *n* = 70). The overall response rate (calculated as the number who provided any biospecimen divided by the number who could be contacted by telephone, were eligible, and were sent a collection kit) was 47%; rates varied widely and were 32% for African-Americans, 39% for Latinas, 40% for Asians, and 82% for non-Latina Whites. The quota of 25 biospecimens per race/ethnic group was achieved for White and Asian women but not for African-Americans and Latinas. The majority of women in the final sample were ≤ age 65, married, had ≥ a high school education, and were above the federal poverty level (Table 2).

**Table 1:** Biospecimen study recruitment outcome for Equality in Breast Cancer Care Study women who were sent a letter of invitation to participate in a biospecimen collection study (*N* = 203)

Recruitment outcome	African-American	Non-Latina White	Latina	Asian	Total
Mailed a letter of invitation	25	50	42	86	203
Deceased	1	0	2	4	7
Ineligible due to other cancer diagnosis or in recurrence and treatment	2	1	0	2	5
Were not sent a collection kit because race/ethnic quota was reached	0	20	0	2	22
Unable to reach by telephone	3	1	7	8	19
Refusals (passive and active)^a^	13	5	20	42	80
Provided any sample (hair or saliva) Hair sample only Saliva sample only Both a hair and saliva sample	6 0 2 4	23 1 0 22	13 0 0 13	28 0 5 23	70 1 7 62
Response rate (% of contacted by telephone, who were sent a collection kit, and eligible)	0.32	0.82	0.39	0.40	0.47

^a^Refusals include women who were reached by phone and refused to provide a biospecimen (hard refusals) or indicated they would think about it and never responded to follow-up calls (soft refusals) or agreed to be sent a collection kit and never responded to follow-up calls (soft refusals)

**Table 2:** Demographic characteristics of Equality in Breast Cancer Care Study women diagnosed between 2006 and 2009, San Francisco Bay Area, who agreed to be recontacted and provided a biospecimen

	All *N* = 70 *n* (%)	Non-Hispanic White *N* = 27 *n* (%)	African-American *N* = 6 *n* (%)	Hispanic *N* = 9 *n* (%)	Asian/Pacific Islander *N* = 28 *n* (%)
Age (at diagnosis) ≤65 >65	43 (6) 27 (39)	16 (59) 11 (41)	3 (50) 3 (50)	4 (44) 5 (56)	20 (71) 8 (29)
Marital status (at diagnosis) MarriedNot married	50 (72) 20 (29)	22 (81) 5 (19)	3 (50) 3 (50)	4 (44) 5 (56)	21 (75) 7 (25)
Highest educational level (at diagnosis) <high school ≥high school	6 (9) 64 (91)	26 (96) 1 (4)	1 (17) 5 (83)	0 (0) 9 (100)	4 (14) 24 (86)
Household poverty status (at diagnosis)^a^ Under federal poverty level Above federal poverty level Unknown	24 (34) 39 (56) 7 (10)	4 (15) 22 (88) 1 (4)	0 (0) 6 (100) 0 (0)	6 (67) 2 (22) 1 (11)	9 (50) 14 (32) 5 (18)

Cells with fewer than five participants are not shown for cancer registry variables (i.e., cancer subtype) due to California Cancer Registry guidelines

^a^Poverty status is calculated using household income (adjusted for household size) to poverty ratio, as defined by the U.S. Department of Health & Human Services

Regarding the quality of samples obtained, a small proportion (15%) of saliva samples appeared to contain food particles even though participants were asked to refrain from eating or drinking coffee prior to collecting saliva. Clear saliva samples provided the best quality gDNA with 260/280 of roughly ~1.7–2.0.

### Optimization of cortisol assay using hair samples

While measuring cortisol extracted from hair overcomes the need for immediate refrigeration and complex sampling regimens required for other types of biospecimens, it is still subject to the limitations of commercially available kits for measuring cortisol. These limitations include the lack of a standardized protocol for use of hair as a biospecimen and a high coefficient of variation in the results obtained. The ALPCO kit used in this study typically yields results with a high coefficient of variation (up to 20% per manufacturer’s specifications) that were slightly improved upon in our study. Specifically, the coefficients of variation for the cortisol levels found in our study ranged from 11 to 16%. These results suggest that the methods we optimized for organic extraction of cortisol from hair, evaporation of the solvent, and resuspension of the extracted materials are suitable.

**Fig. 1:**
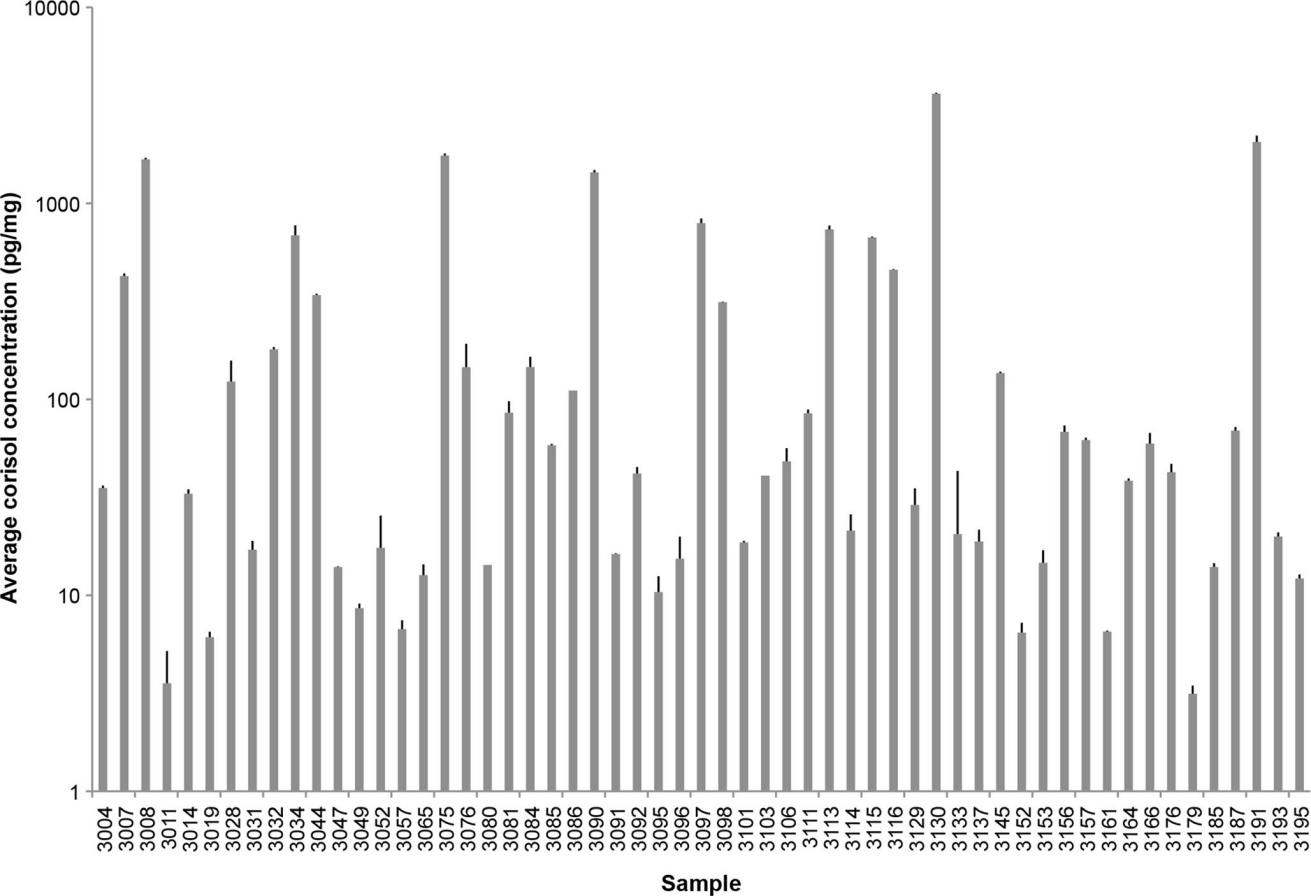
Cortisol levels in hair collected from EBCC participants. Extracted cortisol from each hair biospecimen was quantified using an ELISA-based kit as described by the manufacturer (ALPCO). Hair sampling and analysis were performed in triplicates from a single extraction. ELISA results were converted in picogram per milligram to account for the initial weight of the hair sample. To reliably measure the high levels of cortisol found in many of our hair samples, PBS resuspensions were subjected to dilution (1:2, 1:4, 1:8) to maintain the linear range of the assay (1–100 ng/mL). Plot was generated using Microsoft Excel

Using our optimized protocols for cortisol extraction, the amount of cortisol measured in the 63 hair samples analyzed in our study ranged from 0.8–3631 pg/mg of hair (Fig. 1). This broad range has been found in several studies of cortisol levels in hair. In fact, in these studies the average concentration of cortisol in healthy controls ranged from 7.7 to 224.9 pg/mg and from 10.8 to 295 pg/mg in individuals with a variety of diseases (Wosu et al. 2013; Russell et al. 2012). A recent study showed similar levels of cortisol in a multiethnic sample (Wosu et al. 2015).

### Optimization of telomere length measurement using saliva

Using gDNA extracted from saliva of sufficient quality (i.e., A260/280 >1.7), we measured the relative telomere lengths of 58 participants by qPCR. We amplified telomere repeats (T) and the hemoglobin gene as the single-copy gene (S) to determine the T/S ratio used for calculating TL as previously described (Cawthon 2002; Lin et al. 2010).

**Fig. 2:**
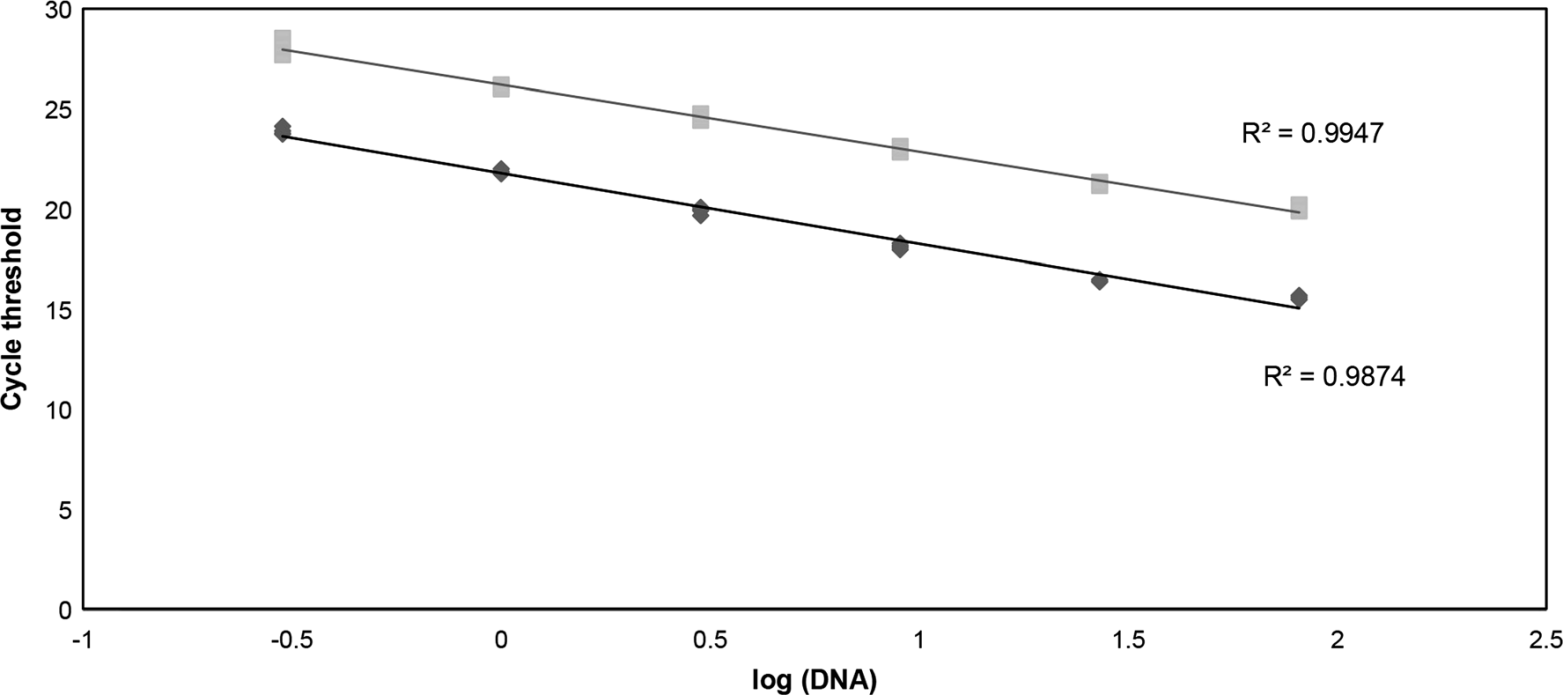
Standard curves used to determine relative telomere length in gDNA obtained from saliva samples. A reference genomic DNA was subjected to a threefold serial dilution (81, 27, 9, 3, 1, and 0.3 ng per well) and aliquoted in triplicates into a 96-well qPCR plate. Both telomere repeats and hemoglobin (reference gene) fluorescent signals were then used to generate plot using Microsoft Excel. *Black diamonds*, telomere repeats (T); *gray squares*, hemoglobin single-copy gene (S)

qPCR conditions were optimized to obtain reliable and reproducible results in experiments performed on different days. To confirm that our results were linear with the amount of DNA added, we generated standard curves to quantify T and S using serial dilutions of a reference genomic DNA (gDNA). Figure 2 shows a typical standard curve with an *R* ^2^ value of 0.99. The coefficient of variation for the T/S ratio was found to be less than 10% between independent replicates (*n* = 3) performed on separate experiments. In addition, each assay included male and female gDNA controls which gave an average inter-assay coefficient of variation for telomere length measurement of 9.1% (*n* = 3).

**Fig. 3:**
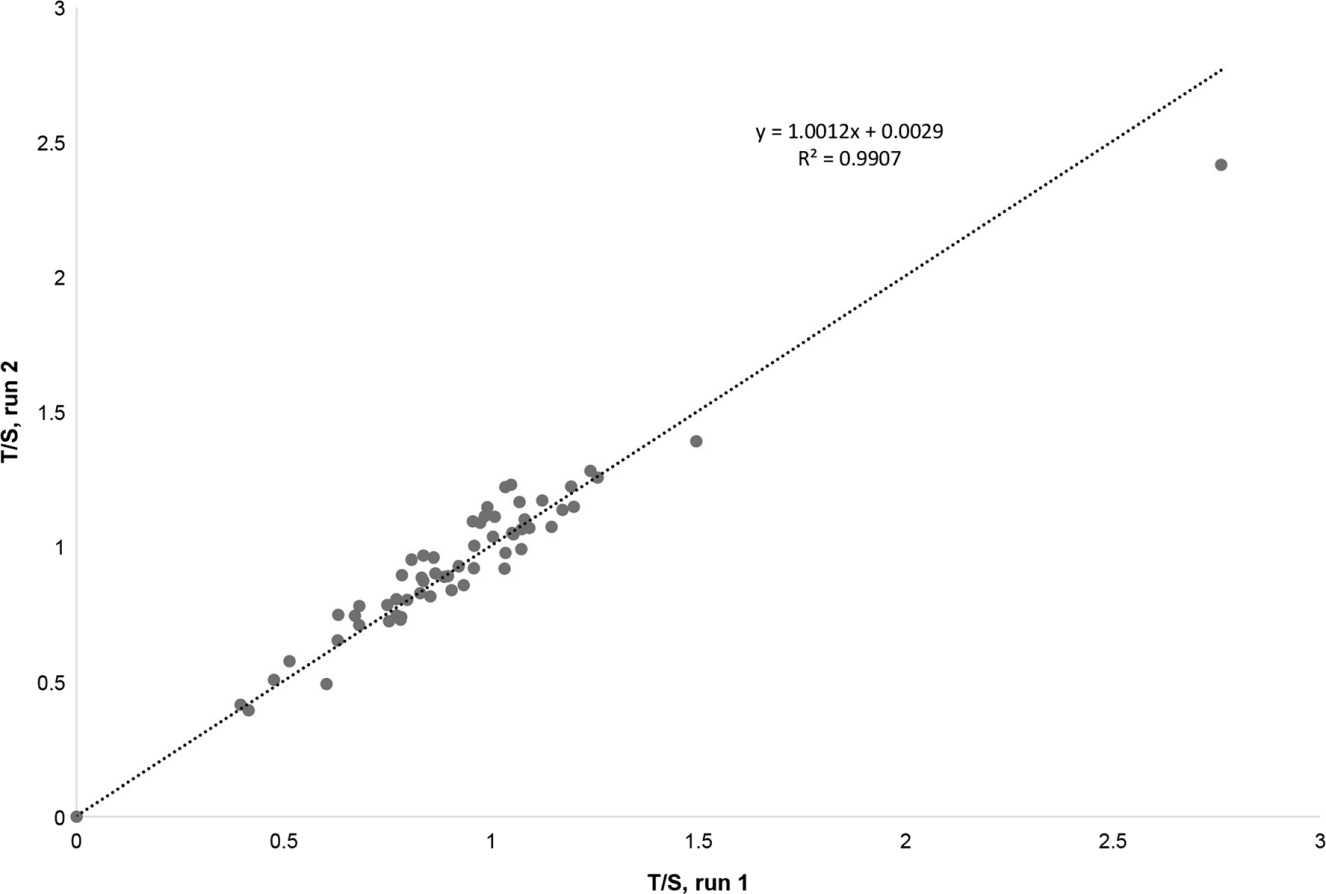
Relative telomere length measurements from two independent runs show reproducibility of qPCR assay. Plot represents the average telomere length obtained from two independent experiments performed using the same well positions. The linear regression equation and correlation coefficient (*R* ^2^) were generated using Microsoft Excel

Having confirmed the reliability of our qPCR approach, the T/S ratios were determined for the gDNA extracted from the self-collected saliva samples. As long as the DNA extracted from the saliva was of sufficient purity (i.e., A260/280 >1.7) the results obtained were reliable with a typical coefficient of variation of less than 10%. DNA samples extracted from ten biospecimens were not included in our investigation due to poor DNA quality (i.e., A260//280 <1.7). We obtained relative telomere length (T/S) measurements for 58 biospecimens and the average T/S ratios determined by the first and second runs were plotted in Fig. 3. The *R* ^2^ value for the comparison of average T/S ratios determined in each run is 0.99 demonstrating the high inter-day reproducibility of our assay. Using this approach, we obtained relative TL measurements (e.g., T/S) ranging from 0.3 to 2.5. These results are similar to those reported by other groups (Aviv et al. 2011; Jodczyk et al. 2014; Lapham et al. 2015).

## Discussion

Our study examined newer, less invasive approaches to biospecimen collection that might be more acceptable to low-income, ethnically diverse populations, and developed molecular biology methods to analyze hair cortisol and saliva telomere length. In this pilot study, we were able to obtain our targeted quota of 25 biospecimens in each group for White and Asian women but not for African-American and Latina women. Nonetheless, response rates for any biospecimen obtained were all above 30%, which we deemed a success given the obstacles observed in prior studies requiring biospecimens among ethnic minorities (Dang et al. 2014). We succeeded in gathering hair and saliva biospecimens and developing biochemical and genetic tools for measuring long-term exposure to cortisol from hair and telomere lengths in DNA extracted from saliva samples. The coefficients of variation for the cortisol levels found in our study (ranging from 11 to 16%) suggest that the methods we used to optimize organic extraction of cortisol from hair, evaporation of the solvent, and resuspension of the extracted materials are suitable. Our study was able to isolate gDNA from self-collected saliva samples from minority breast cancer survivors and optimized laboratory conditions that reliably measured telomere length in DNA samples with an inter-assay coefficient of variation of less than 10%. Thus, these less invasive and practical self-collection methods and analytic procedures may facilitate future research on the effects of exposure to stressful stimuli on cortisol levels and telomere length among ethnically diverse breast cancer survivors.

Of eligible women contacted by telephone who were sent a biospecimen collection kit, our response rates for any biospecimen collected ranged from a high of 82% for White women to a low of 32% for African-American women. While these response rates are modest in terms of recruitment to biomedical research, they are significant with respect to biospecimen donation given the inability of some investigators to collect samples from minority populations in prior studies (Dang et al. 2014). We were unable to compare our response rates by race/ethnicity with other studies collecting similar biospecimens. Similar studies have not reported biospecimen collection response rates by race/ethnicity and only a few studies have used hair as a biospecimen for measuring cortisol. In the few studies that reported the use of hair for cortisol measurements, the response rates for different ethnic groups is not reported (O’Brien et al. 2013; Wosu et al. 2015), as is the case for studies using saliva as a biospecimen (e.g., Lapham et al. 2015; Theall et al. 2013). Our tailored recruitment materials and simplified self-collection approaches allowed us to collect biospecimens from ethnically diverse minority groups who often refuse to donate (Dang et al. 2014) and indicated areas for improvement of recruitment and collection practices.

To engage ethnic minorities in biomedical research, it has been documented that “one size does not fit all” (Umutyan et al. 2008). A primary concern in the use of biospecimen donation for genetic studies is a lack of the public’s understanding of genetics and genetic research (Streicher et al. 2011) and a reluctance to donate tissue and/or visit a medical facility for biospecimen collection (Dang et al. 2014). Previous work has shown that the use of written materials and follow-up phone calls that are in-language and supplemented with verbal explanations by phone increases participation in research among diverse populations (Ramirez et al. 2008). As recommended, we used a linguistically, culturally, and educationally tailored recruitment strategy and in-home self-collection protocol for biospecimen donation. We provided participants with low-literacy instructions for in-home self-collection of hair and saliva samples, supplemented with instructive diagrams. Furthermore, our recruiters spent a substantial amount of time on the telephone answering biological questions about the nature of biospecimens and what they would be used for. We overcame barriers to biospecimen donation by answering sensitively questions during follow-up phone calls to allay fears and suspicions about genetic studies. Furthermore, we used less invasive and less costly biospecimen collection methods, hair and saliva instead of serum or tissue, and self-collection, rather than requiring a visit to a laboratory or a home visit by a phlebotomist. Use of such strategies is highly recommended when attempting to secure biospecimens from ethnically and socioeconomically diverse populations due to lack of familiarity with such studies and potential mistrust regarding use of genetic information.

We gained information from this pilot study on ways to improve on our collection methods. Collection of hair was more straightforward for participants than collection of saliva; all hair samples were of sufficient quality for measuring long-term exposure to cortisol. However, more participants chose not to donate hair than saliva, and this was especially true for African-American women. Future work is needed to better understand barriers to hair donation and to identify alternative methods for collecting suitable biospecimens. Self-collection of saliva samples resulted in some samples containing impurities that affected gDNA quality. These results suggest that participants failed to adhere to the written directions for collection of saliva, e.g., they ate a snack after brushing their teeth at bedtime and before collecting their saliva first thing in the morning. Thus, clarifying and reinforcing the importance of the recommended procedures for collection of specimens may be needed. In the future, the directions for collection of saliva should explicitly ask participants to refrain from eating after going to bed and include explicit directions for using a mouthwash (including a mouthwash sample in the kit) prior to providing the saliva sample. Additionally, a link to an online video documenting collection protocols for both hair and saliva might enhance adherence.

Measuring cortisol from hair (as opposed to other biospecimens such as blood, saliva, or urine) offers experimental and biological advantages. Cortisol remains stable in hair for up to 3 months (Russell et al. 2012), facilitating self-collection and mailing of samples, as well as prolonged sample storage at room temperature. Additionally, multiple cortisol measurements can be conducted from a single hair sample alleviating the stress and burden caused by the need for multiple, more invasive sample collections to account for daily variation in blood, urine, and saliva (Chen et al. 2013). In fact, cortisol measured from hair provides a record of long-term exposure to cortisol because hair grows about 1 cm per month. Thus, the snipped hair from the scalp end of the samples collected in our study recorded cortisol exposure for the last several weeks or longer. For already stressed socially disadvantaged populations, these relatively simpler biospecimen collection features may make participation in such studies more acceptable, once attitudinal and informational barriers are addressed.

The determination of cortisol levels using hair as a biospecimen has received a great deal of attention by many scientific groups as a useful biomarker of chronic stress. However, the procedures used to determine cortisol levels from this biospecimen vary across the scientific research community. To date, the scientific literature does not provide standardized guidelines for processing of hair, cortisol extraction, evaporation of solvent, and resuspension of extracted materials (Albar et al. 2013). The lack of these guidelines could create variability in reported cortisol measurements across studies. Nonetheless, measurement of cortisol levels from hair offers distinct advantages over the use of other biospecimens for this type of analysis. Our biochemical approach yielded reproducible measurements of cortisol levels, but as documented by others, the coefficients of variation were large using the ELISA-based approach, indicating a need for better methods to measure cortisol. Additionally, the range of values obtained for all samples was broad and difficult to interpret given the lack of codified values for low and high cortisol. While all of the hair samples were of sufficient quality for use in the cortisol assay, the coefficients of variation for the results obtained were high. These coefficients ranged from 11 to 16%, and were below the 20% cutoff recommended by the manufacturer (ALPCO) of the ELISA-based assay used in this study. Our future work is aimed at optimizing a mass spectrometry approach for measuring cortisol levels in hair. Our preliminary results show that this approach yields coefficients of variation <5%, and that it specifically measures just cortisol (not other steroids). In fact, previous investigators have noted that cross reactivity of other steroids with the antibodies used in the ELISA-based kits results in low specificity of the results obtained (Gao et al. 2013, Xing et al. 2013).

There is evidence that gDNA isolated from saliva samples is a valid source of genetic material for measuring telomere length. Strong and valid correlations have been found between TL determined from blood and saliva samples from the same individual (Mitchell et al. 2014). In fact, positive correlations between TL measured from gDNA that is isolated from blood, skin, skeletal muscle, and subcutaneous fat have been documented (Daniali et al. 2013). These findings indicate that despite vastly different developmental lineages and rates of proliferation, somatic tissues collected from the same individual show similar rates of telomere length shortening. However, results obtained for relative TL are highly specific due to the nature of the qPCR approach. The DNA extracted from 58/69 (84%) saliva samples allowed us to specifically quantify telomere repeats (T) across all chromosomes relative to the amount of the hemoglobin gene that exists as a single copy (S) in the genome. The resulting T/S ratio was then used to determine relative telomere length (TL). This length is considered a robust indicator of biological age and overall health (Sanders and Newman 2013), as well as a suitable biomarker for life stress (Epel et al. 2004). Our genetic approach to measuring telomere lengths showed coefficients of variation (<10%) suggesting that our results are reproducible.

The results of our pilot study are subject to several important limitations, including a small sample size. Moreover, we did not include samples from comparison groups such as healthy individuals and/or individuals with known stress exposures. This study also does not provide a longitudinal trajectory of changes in cortisol levels and relative TL, but rather, we established the basis for a standardized protocol to collect and analyze biospecimens from ethnic minority populations to measure these biomarkers of chronic stress. Despite these limitations, our results show that we can engage ethnic minority populations in self-collection and donation of hair and saliva samples for relative telomere length and cortisol measurement and suggest ways to further improve response rates among ethnically diverse groups. We hope that such methods will facilitate future studies to gain a better understanding of the complex relationships between chronic stress due to social disadvantage and biological mechanisms that underlie health outcomes of ethnically diverse populations.

In 2014, racial/ethnic minorities made up nearly 40% of the US population (United States Census Bureau Quick Facts 2016) and their under-representation in biomedical studies has been described as a missed scientific opportunity to fully understand the factors that lead to disease or health (Oh et al. 2015). Furthermore, the inability to collect biospecimens limits the promise of personalized medicine to improve diagnostic tests and treatment for all populations (Dang et al. 2014). This study demonstrates the feasibility of biospecimen collection across racial/ethnic groups, although clearly, more work to promote participation in biomedical research among ethnically diverse populations is needed.
